# Impact of Immunonutrition on T Cell Activation: A Randomized Control Study in Cardiac Surgery Patients

**DOI:** 10.15388/Amed.2021.28.2.16

**Published:** 2021-12-16

**Authors:** Marija Svetikienė, Dainius Trybė, Marius Strioga, Jevgenija Vėželienė, Viktoras Isajevas, Radvilė Malickaitė, Laimutė Jurgauskienė, Donata Ringaitienė, Mindaugas Šerpytis, Jūratė Šipylaitė

**Affiliations:** Department of Anaesthesiology and Intensive Care, Institute of Clinical Medicine, Faculty of Medicine, Vilnius University Vilnius University Hospital Santaros Klinikos, Vilnius, Lithuania; Faculty of Medicine, Vilnius University, Vilnius, Lithuania; Faculty of Medicine, Vilnius University, Vilnius, Lithuania; Vilnius University Hospital Santaros Klinikos, Vilnius, Lithuania; Department of Anaesthesiology and Intensive Care, Institute of Clinical Medicine, Faculty of Medicine, Vilnius University Vilnius University Hospital Santaros Klinikos, Vilnius, Lithuania; Clinic of Cardiac and Vascular Diseases, Institute of Clinical Medicine, Faculty of Medicine, Vilnius University, Vilnius, Lithuania; Clinic of Cardiac and Vascular Diseases, Institute of Clinical Medicine, Faculty of Medicine, Vilnius University, Vilnius, Lithuania; Department of Anaesthesiology and Intensive Care, Institute of Clinical Medicine, Faculty of Medicine, Vilnius University Vilnius University Hospital Santaros Klinikos, Vilnius, Lithuania; Vilnius University Hospital Santaros Klinikos, Vilnius, Lithuania; Department of Anaesthesiology and Intensive Care, Institute of Clinical Medicine, Faculty of Medicine, Vilnius University Vilnius University Hospital Santaros Klinikos, Vilnius, Lithuania

**Keywords:** cardiac surgery, immunonutrition, glutamine, cytokine, CD4+, CD8+, CD69+

## Abstract

**Background.:**

Cardiac surgery provokes an intense inflammatory response that can cause an immunosuppressive state and adverse postoperative outcomes. We recently showed that postoperative immunonutrition with glutamine in “fragile” low-risk cardiac surgery patients was associated with a significantly increased level of CD3+ and CD4+ T cells. In order to clarify the biological relevance and clinical importance of these findings, we investigated whether an increase in the CD4+ T cell level was caused by changes in the systemic inflammatory response (caused by surgery or infection) and if it was associated with their activation status.

**Methods.:**

A randomized control study of low operative risk but “fragile” cardiac surgery patients was performed. Patients were randomized into immunonutrition (IN) and control groups (C). The IN group received normal daily meals plus special immune nutrients for 5 days postoperatively, while the C group received only normal daily meals. Laboratory parameters were investigated before surgery and on the sixth postoperative day and the groups were compared accordingly. The expression of the CD69+ marker was investigated to determine T cell activation status. Serum concentrations of cytokines (interleukin-10 (IL-10), tumor necrosis factor α (TNF-α) and interleukin-6 (IL-6)) and C-reactive protein (CRP) were determined to assess the systemic inflammatory response, while procalcitonin (PCT) levels were evaluated to confirm or deny possible bacterial infection.

**Results.:**

Fifty-five patients were enrolled in the study. Twenty-seven (49.1%) were randomized in the IN group. Results show that on the sixth postoperative day, the CD4+CD69+ and CD8+CD69+ counts did not differ between the IN and C groups, accordingly 0.25 [0.16–0.50] vs 0.22 [0.13-0.41], p=0.578 and 0.13 [0.06–0.3] vs 0.09 [0.05–0.14], p=0.178. Also, statistically significant differences were not observed in the cytokine levels (IN and C groups: TNF-α 8.13 [7.32–10.31] vs 8.78 [7.65–11.2], p=0.300; IL-6 14.65 [9.28–18.95] vs 12.25 [8.55–22.50], p=0.786; IL-10 5.0 [5.0–5.0] vs 5.0 [5.0–5.0], p=0.343 respectively), which imply that an elevated T cell count is not associated with the systemic inflammatory response. Also, PCT (IN and C groups: 0.03 [0.01–0.09] vs 0.05 [0.03–0.08], p=0.352) and CRP (IN and C groups 62.7 [34.2–106.0] vs 63.7 [32.9–91.0], p=0.840) levels did not differ between the two groups. Moreover, low levels of PCT indicated that the increase in T cell count was not determined by bacterial infection.

**Conclusions.:**

Our findings showed that CD4+ T cell levels were associated with neither the systemic inflammatory response nor bacterial infection. Secondly, increases in T cells are not accompanied by their activation status. These results suggest a hypothesis that a higher postoperative T cell concentration may be associated with postoperative immunonutrition in low-risk cardiac surgery patients with intact cellular vitality, i.e. “fragile”. However, immunonutrition alone did not affect T cell activation status.

## Abstract (lt)

**Imunomitybos poveikis T ląstelių aktyvacijai: atsitiktinių imčių tyrimas kardiochirurginių ligonių grupėje:**

**Santrauka. *****Apžvalga.*** Kardiochirurginiams pacientams širdies operacijos metu yra būdingas ryškus sisteminio uždegiminio atsako sindromas, kuris gali lemti kompensacinę imunosupresinę būklę ir blogesnes pooperacines baigtis. Ankstesniame straipsnyje nustatėme, kad „trapių“, bet mažos operacinės rizikos pacientų pooperacinė imunomityba glutamino pagrindu buvo susijusi su padidėjusiu cirkuliuojančių CD3+ ir CD4+ T ląstelių skaičiumi. Šiuo tyrimu bandėme išsiaiškinti, ar CD4+ T ląstelių padidėjimas buvo nulemtas sisteminio uždegiminio atsako sindromo (kurį galėjo sukelti chirurginė intervencija ar infekcija) ir ar šis padidėjimas buvo susijęs su T ląstelių aktyvacija.

***Metodai.*** Buvo atliktas randomizuotas atsitiktinių imčių tyrimas su mažos operacinės rizikos „trapiais“ kardiochirurginiais ligoniais. Pacientai suskirstyti į imunomitybos (IM) ir kontrolinę (K) grupes. IM grupės pacientai penkias pooperacines dienas buvo maitinami įprastu maistu ir gaudavo specialų peroralinį imunomišinį glutamino pagrindu, o K grupė maitinta tik įprastu maistu. Laboratoriniai tyrimai buvo atliekami prieš operaciją ir šeštą pooperacinę dieną. T ląstelių aktyvacijai nustatyti buvo naudojamas CD69+ žymuo. Sisteminiam uždegiminiam atsakui įvertinti tirtos citokinų (interleukino 10 (IL-10), tumoro nekrozės faktoriaus α (TNF-α), interleukino 6 (IL-6)) ir C reaktyviojo baltymo (CRB) koncentracijos. Galimoms bakterinėms infekcijoms paneigti ar patvirtinti naudotas prokalcitoninas.

***Rezultatai.*** Mūsų tyrimo imtis – penkiasdešimt penki ligoniai. Dvidešimt septyni ligoniai (49,1 %) buvo įtraukti į IM grupę. Šeštą pooperacinę dieną CD4+CD69+ ir CD8+CD69+ ląstelių koncentracija nesiskyrė tarp grupių (0,25 [0,16–0,50] vs 0,22 [0,13–0,41], p = 0,578 ir 0,13 [0,06–0,3] vs 0,09 [0,05–0,14], p = 0,178). Citokinų koncentracija tarp grupių taip pat nesiskyrė (IM ir K grupių atitinkamai: TNF-α 8,13 [7,32–10,31] vs 8,78 [7,65–11,2], p = 0,300; IL-6 14,65 [9,28–18,95] vs 12,25 [8,55–22,50], p = 0,786; IL-10 5,0 [5,0–5,0] vs 5.0 [5,0–5,0], p = 0,343). Tai rodo, kad padidėjęs T ląstelių skaičius nėra susijęs su sisteminiu uždegiminiu atsaku. Prokalcitonino ir CRB koncentracijos taip pat nesiskyrė tarp grupių (IM ir K grupių atitinkamai: prokalcitoninas 0,03 [0,01–0,09] vs 0,05 [0,03–0,08], p = 0,352; CRB 62,7 [34,2–106,0] vs 63,7 [32,9–91,0], p = 0,840). Nedidelės prokalcitonino koncentracijos rodo, kad T ląstelių skaičiaus padidėjimas nebuvo nulemtas bakterinės infekcijos.

***Išvados.*** Remiantis šio tyrimo rezultatais galima teigti, kad CD4+ T ląstelių skaičiaus padidėjimas nebuvo susijęs su sisteminio uždegiminio atsako sindromu ar bakterine infekcija. Taip pat T ląstelių skaičiaus padidėjimas nebuvo lydimas didesnės jų aktyvacijos. Manome, kad šis T ląstelių skaičiaus padidėjimas mažos operacinės rizikos kardiochirurginiams pacientams, turintiems sumažėjusį ląstelių gyvybingumą, t. y. „trapiems“, gali būti susijęs su taikyta pooperacine imunomityba.

**Raktažodžiai:** širdies chirurgija, imunomityba, glutaminas, citokinai, CD4+, CD8+, CD69+

## Introduction

Cardiac surgery provokes an intense inflammatory response due to multifactorial causes, including mechanical surgical injury, blood loss and transfusion, ischaemia-reperfusion syndrome and blood contact with artificial surfaces during cardiopulmonary bypass (CPB) [1,2,3]. Stimulation of pro-inflammatory cytokines, such as interleukin-1 (IL-1), tumor necrosis factor α (TNF-α) and interleukin-6 (IL-6), guide the outcome of T cell activation and differentiation [4]. Earlier studies have demonstrated that increases in pro-inflammatory cytokines are associated with adverse outcomes following cardiac surgery [5,6,7]. The release of pro-inflammatory mediators is balanced by a phase of anti-inflammatory cytokines, such as interleukin-10 (IL-10), whose prolonged secretion can cause transient immunosuppression. These perturbations of the immune system are well described in cardiac surgery [2,5,8,9,10] and are associated with organ dysfunction, prolonged hospital stays and poorer cardiac surgery outcomes.

Due to the progress of interventional cardiology, cardiac surgery patients have become older with abundant comorbidities. The term “frailty” or “fragility” has become very relevant in cardiac surgery.

“Fragility” is considered to be a syndrome characterized by excessive vulnerability to stressors with reduced ability to maintain or restore homeostasis after a destabilizing event [11]. This concept is associated with an increased risk of disability and morbidity after surgery, especially after major surgeries, such as cardiac surgery [12].

Clinical studies have shown that the use of supplementary nutrients (such as arginine or glutamine) has a positive effect on the immune system [13]. Administration of glutamine in major surgery reduces inflammatory cytokines and increases lymphocyte concentrations [14]. Studies on abdominal, gynaecological, and oncological surgeries have demonstrated that immunonutrition has an effect on cellular immunity by increasing the total count of lymphocytes. These effects are more prominent in patients who are already malnourished and “frail” before surgery. Our study recently revealed that postoperative supplementation of “fragile” low-risk cardiac surgery patients with immunonutrition for 5 days postoperatively was associated with significantly increased levels of both CD3+ and CD4+ T cells [15]. Although the biological relevance and clinical importance of these findings remains unclear, we conducted additional research. 

In this article, we investigated whether an increase in CD4+ T cell levels could be caused by factors other than immunonutrition. This change in CD4+ T cell count may have been due to a pronounced systemic inflammatory response that may have been induced by surgery or bacterial infection. Secondly, it was investigated whether the increase in CD4+ T cells is related to their activation status as assessed by expression of the early activation marker CD69+.

## Methods

This randomized controlled study was approved by the Vilnius Regional Research Ethics Committee. Informed consent and permission to release information were obtained from all patients. The study was conducted between February 2015 and June 2017 in a tertiary referral university hospital. The first data were published in our article [15]. Additional tests were performed on blood samples and examined expression of the CD69+ marker on T cells. The study was registered with ClinicalTrials.gov (Identifier: NCT04047095).

### Patient population

Patients undergoing elective cardiac surgery were included in the study. All patients were screened for their eligibility to participate in the study according to the inclusion and exclusion criteria. Inclusion criteria were used to generate low operative risk and “fragile” patient groups. The exclusion process was carried out throughout the trial by applying preoperative, operative and postoperative criteria. The inclusion and exclusion criteria are presented in Table 1.

**Table 1.** Selection criteria

**Inclusion criteria**
Elective cardiac surgery with cardiopulmonary bypass (CPB): • coronary artery bypass grafting (CABG); • aortic valve replacement; • mitral valve replacement; • mitral valve repair; • tricuspid valve repair; • combined operations (CABG and valve surgery). Phase angle < 5.5°.
**Exclusion criteria**
Preoperative: • previous cardiac surgery; • left ventricle ejection fraction < 40%; • use of preoperative intra-aortic balloon pump (IABP); • critical preoperative state; • pulmonary artery mean pressure > 55 mmHg; • diagnosis of infectious endocarditis; • pacemaker. Operative: • complicated intraoperative course (unplanned intervention or low cardiac output syndrome in the operating theatre: failure to wean from CPB, intraoperative insertion of IABP or infusion of two or more inotropic medications with a cumulative dose of 0.2 mcg/kg/min); • surgery time > 6 h; • unplanned intervention. Postoperative: • disturbance of dietary rules.

### Groups

Patients were randomly divided into two groups: intervention (immunonutrition (IN)) and control (C) groups. Patients in the IN group received normal daily meals plus one sachet three times a day of immunonutrients (“Glutamine Plus” by Fresenius Kabi) for 5 days after the surgery, starting in the first 24 h postoperatively. One sachet (22.4 g) of “Glutamine Plus” contained glutamine 10 g, carbohydrate 10 g, b-carotene 1.7 mg, vitamin E 83 mg, vitamin C 250 mg, zinc 3.4 mg, selenium 50 mg and fibre 1.2 g. The cumulative daily dose of immunonutrients was 67.2 g (30 g of glutamine). The C group was provided with normal daily meals. The patients were excluded from the study if they had failed to take all of the prescribed immunonutrients.

### Sample collection

All patients underwent bioelectrical impedance analysis (BIA) before they were included in the study. This was used to form a “fragile” patient group and was performed 1 day before the surgery. BIA provided a 50 kHz segmental phase angle (PhA) measurement and patients were included in the study if the PhA was < 5.5⁰.

Patients’ demographic data, comorbidities, preoperative and postoperative laboratory and instrumental parameters were gathered from medical records after the inclusion process. 

To determine the differences between the groups, blood samples were taken twice, on the morning before the surgery and on the sixth postoperative day. The term of 5 postoperative days was chosen because studies in cardiac surgery patients have shown that postoperative immunosuppression is observed at the level of nonspecific immunity up to 5 days, then the changes return to normal. However, a decrease in the number of cells of cellular immunity and changes in balance between subgroups can persist more than 5 days [16,17].

The systemic inflammatory response was evaluated by measuring serum concentrations of cytokines (TNF-α, IL-6 and IL-10) and C-reactive protein (CRP), whereas activation of T cells was assessed by the expression of the classical T cell activation marker CD69+ [18,19]. The count of CD69+ T cells and concentrations of cytokines were subsequently determined from blood samples. Samples for cytokine measurement were stored at –80 °C until examination. Additionally, procalcitonin (PCT) was measured as a marker of bacterial infection.

Some patients were eliminated from the study at the interleukins survey stage due to the lack of frozen plasma samples to determine interleukin concentrations.

### CD69+ marker assessment

Peripheral blood mononuclear cells (PBMCs) were isolated under sterile conditions using density gradient centrifugation through lymphocyte isolation suspension (Lymphoprep^TM^, Axies-Shield Poc AS, Norway). After the washing step, the PBMCs were suspended in RPMI 1640 medium (RPMI 1640 Medium, HyClone Laboratories, USA) with 20% newborn calf serum (Life Technologies, USA) supplemented with penicillin-streptomycin solution (Biological Industries, Israel) and seeded in 10 cm^2^ surface activated growth area TPP tissue culture tubes (TPP Techno Plastic Products AG, Switzerland) for phytohemagglutinin (PHA-P, Sigma-Aldrich) stimulation testing (PHA concentration 10 µg/ml). Samples were cultured at 37 °C in a humidified 5% CO_2 _cell culture incubator. After 18 h of incubation, samples were stained using the monoclonal antibodies CD3PerCP / CD14PE / CD45FITC, CD3PerCP / γ1PE / γ2aFITC, CD3PerCP / CD4PE / CD69FITC and CD3PerCP / CD8PE / CD69FITC (BD Biosciences, San Jose, CA, USA). Antigen expression was analyzed using a FACSCalibur flow cytometer (BD, USA) with CellQuestPro analysis software (BD Biosciences, San Jose, CA, USA). Cells were sequentially gated on lymphocytes (based on side scatter (SSC) vs. CD3PerCP dot plot), after which activation marker CD69+ percentages on the CD4+ and CD8+ subpopulations were determined. 

### Inflammation marker and interleukins assessment

After thawing, all serum samples were tested simultaneously. The prohormone of calcitonin, PCT, was measured on an ADVIA Centaur XP random access analyzer (Siemens Healthcare Diagnostics) using the ADVIA Centaur® BRAHMS PCT assay (the assay is a two-site sandwich immunoassay using direct chemiluminescent technology that uses three mouse monoclonal antibodies specific for PCT. A direct relationship exists between the amount of PCT present in the patient sample and the number of relative light units (RLUs) detected by the system). Quantitative measurements of TNF-α, IL-6 and IL-10 were performed on the IMMULITE 1000 random access immunoassay system using TNF-α, IL-6 and IL-10 test kits (all Siemens Healthcare Diagnostics) and a solid-phase chemiluminescent immunometric assay.

### Phase angle

One of the inclusion criteria was the status of patients’ cells. To determine body composition and estimate cell vitality objectively, we used BIA analysis, especially one parameter – phase angle (PhA) [20]. BIA is a simple, noninvasive technique that estimates body composition by measuring the opposition (impedance) to an applied external current through the body. Impedance consists of two components: resistance (reflects the flow of altering current through intra- and extracellular solutions) and reactance (reflects cellular membrane capacity). PhA is the ratio between reactance and resistance which reflects the status of the cell membrane and hydration (i.e., cellular vitality) [21,22]. Patients with a low PhA are more likely to be “fragile” and malnourished [23]. There are several studies confirming that BIA PhA can be used in “fragility” diagnostics [24,25] and is associated with adverse postoperative outcomes. In fact, after many attempts to diagnose “fragility” [26,27,28,29,30,31], no single method has emerged as a “gold standard” [32]. Also, there is no validated PhA value reflecting the vulnerability of cardiac surgery patients. Tanaka et al. [20], in a study with noncardiac surgery patients, investigated that, at a BIA PhA value < 5.5°, “fragility” could be determined by other known methods, such as the Fried Frailty Index and Rockwood Clinical Frailty Scale. Another study on cardiac surgery patients determined that a PhA < 5.38⁰ is associated with undernutrition and adverse clinical outcomes after cardiac surgery [33]. Relying upon these studies, we adjusted it to 5.5° and set it as the cut-off.

The procedure is quite clear and simple. Before the analysis, the patients assumed a lying posture for 10 min. During the analysis, the patients were in a supine position with the arms abducted 15⁰ from the trunk and the legs spread apart at shoulder width. The analysis was performed using eight electrodes placed on both hands (on the thumb and middle finger) and between the patient’s ankle bones and heels.

### Statistical analysis

The sample size was determined on the basis of available resources of clinical data and clinical practice. The formula used to determine the sample size was: *N *= (*z*_1–_*_α_*_/_ *s_d_*/*d̄*)^2^, *α* = 0.05, *z*_1–_*_α_*_/2_=1.96, where *d̄* = the difference of measurements means and *s_d_* = the standard deviation of the differences (for the quantitative variables). Each variable was examined separately. According to the formula, a reliable sample size for the results consisted of at least 12 patients for each group.

The selection process for randomization was computer generated and provided by the statistician. The success of the randomization was tested by comparing the intervention and control groups for differences in demographics, comorbidities and laboratory results, including immunological, before the intervention. The statistical analysis was performed using IBM SPSS V.21 software. Baseline characteristics were defined by descriptive statistics. Continuous variables were described as means ± standard deviations (SDs) or medians (interquartile ranges [IQRs]), and categorical variables were described as n (%). The normality of variable distributions was tested using the Kolmogorov–Smirnov test. Student’s t-test and the Mann–Whitney U test were used to compare values according to their normality of distribution. The Pearson chi-square test or Fisher’s exact test was used for comparison of categorical variables. The data were considered to be statistically significant if p was < 0.05.

## Results

### Baseline characteristics

A total of 604 were eligible for the study. However, 517 patients were excluded from the study for not meeting the inclusion criteria, with the most common reason being PhA > 5.5°. In all, 87 patients were included in the study and underwent randomization – 44 patients were assigned to the IN group and 43 patients were assigned to the C group. Additionally, 32 patients were excluded throughout the study. The most common reason for postoperative exclusion was the disturbance of dietary intake (16 cases: 13 in the IN group and three in the C group). Other reasons for post-operative exclusion were unplanned interventions (four cases: one in the IN group and three in the C group), complication of low cardiac output syndrome (six cases: two in the IN group and four in the C group) and surgery time > 6 h (six cases: one in the IN group and five in the C group). Fiftyfive patients finished the study – 27 (49.1%) in the IN group and 28 in the C group. The mean score on the EuroSCORE II was 1.99 ± 0.83%, and the median value of the PhA was 5.18⁰ [4,86–5,32]. Based on these, low operative risk and “fragile” patient groups were formed. The mean age of the cohort was 69.7 ± 6.3 years, and about half (49.1%) of the sample were women. Basic demographic and clinical data on both patient groups are shown in Table 2. There were no statistical differences between the groups before the intervention.

**Table 2.** Baseline demographics and clinical characteristics of patients

	IN group	C group	p
**Demographic profile**			
Age (years)	68.3 ± 6.9	71.0 ± 5.4	0.112
Sex:			
Male, n (%)	14 (51.9)	14 (50.0)	0.891
Female, n (%)	13 (48.1)	14 (50.0)	
Body mass index (kg/m^2^)	28.6 ± 5.1	27.8 ± 4.5	0.550
Phase angle (0)	5.2 [5.04–5.32]	5.11 [4.72–5.30]	0.117
**Comorbidities**			
NYHA classification:			>0.05
Class II, n (%)	3 (11.1)	0 (0)	
Class III, n (%)	24 (88.9)	28 (100.0)	
Class I and IV, n (%)	0 (0)	0 (0)	
ASA physical status			
ASA III, n (%)	27 (100.0)	28 (100.0)	>0.05
CCI	3 [2.00–4.00]	3 [3.00–3.75]	0.570
Arterial hypertension, n (%)	23 (85.2)	26 (92.9)	0.362
Renal failure, n (%)	2 (7.4)	1 (3.6)	0.531
Myocardial infarction, n (%)	9 (33.3)	5 (17.9)	0.188
Diabetes, n (%)	7 (25.9)	7 (25.0)	0.937
**Operative characteristics**			
EuroScore II (%)	1.97 ± 0.8	1.99 ± 0.8	0.918
Operation type:			>0.05
CABG, n (%)	17 (63.0)	18 (64.3)	
Aortic valve, n (%)	4 (14.8)	4 (14.3)	
Mitral valve, n (%)	3 (11.1)	1 (3.6)
Tricuspid valve, n (%)	1 (3.7)	0 (0)
Combined, n (%)	3 (11.1)	5 (17.8)
Aortic cross-clamp time (min)	75.5 ± 29.3	75.2 ± 25.4	0.966
CPB time (min)	112.6 ± 39.3	109.2 ± 34.2	0.735
Operation time (min)	257.4 ± 73.5	258.75 ± 62.4	0.944

NYHA – New York Heart Association; CABG – coronary artery bypass grafting; ASA – American Society of Anesthesiologists; CCI – Charlson comorbidity index

Mean value and standard deviation (mean ± SD); median and interquartile range (median [IQR]); p value

### CD69+ marker

The absolute counts (calculated as cell number *10^9^/l) of CD4+CD69+ and CD8+CD69+ lymphocytes on the sixth postoperative day in the IN group and the C group were as follows: 0.25 [0.16–0.50] vs. 0.22 [0.13–0.41] and 0.13 [0.06–0.26] vs. 0.09 [0.05–0.14], respectively. However, there was no significant difference between the groups after the intervention (CD4+CD69+ p = 0.578, CD8+CD69+ p = 0.178) (Table 3).

**Table 3.** Pre- and postoperative immune cell indicators

	Preoperative			Postoperative		
	IN group	C group	p	IN group	C group	p
CD3+ T cells (*10^9^/l)	1.44 ± 0.61	1.15 ± 0.62	0.093	1.42 ± 0.49	1.12 ± 0.56	0.035
CD4+ T cells (*10^9^/l)	0.95 ± 0.39	0.76 ± 0.41	0.091	1.02 ± 0.36	0.80 ± 0.43	0.048
CD8+ T cells (*10^9^/l)	0.48 ± 0.28	0.38 ± 0.29	0.191	0.40 ± 0.21	0.30 ± 0.18	0.066
CD4+CD69+ T cells (*10^9^/l)	0.40 ± 0.31	0.30 ± 0.25	0.304	0.25 [0.16–0.50]	0.22 [0.13–0.41]	0.578
CD4+CD69+ T cells (%)	21.1 ± 11.85	20.7 ± 9.4	0.876	17.4 ± 11.1	18.0 ± 9.7	0.813
CD8+CD69+ T cells (*10^9^/l)	0.31 ± 0.46	0.15 ± 0.15	0.121	0.13 [0.06–0.3]	0.09 [0.05–0.14]	0.178
CD8+CD69+ T cells (%)	12.3 [5.4–17.1]	7.4 [5.53–10.75]	0.210	7.1 [3.2–14.7]	6.45 [3.75–9.07]	0.578

Mean value and standard deviation (mean ± SD); median and interquartile range (median [IQR]); p value

### Cytokines and procalcitonin

The cytokine levels were not measured in 11 patients due to an insufficient amount of frozen plasma to determine cytokine concentrations. IL-6, IL-10, TNF-α and PCT concentrations were measured in the samples taken before surgery and on the sixth postoperative day in 44 patients – 24 (54.5%) in the IN group (mean age 68.2 ± 7.1 years) and 20 in the C group (mean age 72.4 ± 4.7 years). No statistically significant differences in the cytokine concentrations between the groups were observed either before or after the intervention (Table 4).

**Table 4.** Pre- and postoperative cytokines (TNF-α, IL-6 and IL-10), PCT and CRP concentrations

	Preoperative			Postoperative		
	IN group	C group	p	IN group	C group	p
PCT (mcg/l)	0.01 [0.01–0.01]	0.01 [0.01–0.01]	0.898	0.03 [0.01–0.09]	0.05 [0.03–0.08]	0.352
CRP (mg/l)	1.87 [0.80–4.70]	1.33 [0.70–3.25]	0.429	62.7 [34.2–106.0]	63.7 [32.9–91.0]	0.840
TNF-α (ng/l)	7.23 ± 3.23	8.03 ± 3.05	0.406	8.13 [7.32–10.31]	8.78 [7.65–11.2]	0.300
IL-6 (ng/l)	3.21 [2.61–4.71]	3.15 [2.43–7.67]	0.588	14.65 [9.28–18.95]	12.25 [8.55–22.50]	0.786
IL-10 (ng/l)	5.0 [5.0–5.0]	5.0 [5.0–5.0]	0.192	5.0 [5.0–5.0]	5.0 [5.0–5.0]	0.343

Mean value and standard deviation (mean ± SD); median and interquartile range (median [IQR]); p value

## Discussion

As we discovered in our previous article, low-risk and “fragile” cardiac surgery patients who were supplemented with glutamine and antioxidants for 5 days postoperatively had a statistically higher counts of both CD3+ and CD4+ T cells. This finding led us to carry out further research in an attempt to highlight the impact of the immunonutrition formula on the immune system in low operative risk cardiac surgery patients with impaired cellular vitality, as measured by BIA.

To determine the operative risk of these patients, we used the EuroSCORE II value, as it is recommended by European Society of Cardiology and European Association for Cardio-Thoracic Surgery [34,35]. The EuroSCORE II is a validated prognostic tool reflecting mortality in cardiac surgery. In our selected cohort, the mean EuroSCORE II value was below 2% [36], which according to the global risk of mortality in cardiac surgery presented in the Society of Thoracic Surgeon’s database was ranked as low risk [37].

In our study, we used an immunonutrition formula composed of antioxidants, microelements, fi-bre, and most importantly glutamine. Glutamine is a nonessential amino acid in the human body that is highly involved in various metabolic processes, including its pivotal role in lymphocyte activation [38,39] as an energy-intensive process [19], especially in stressful conditions (such as cardiac surgery). In this case, immune system cells, such as lymphocytes, neutrophils, and macrophages, use glutamine in large quantities, often even higher than glucose in such conditions and the stores of glutamine are often depleted [40,41,42,43]. Studies with cardiac surgery patients showed that postoperative glutamine concentrations were significantly lower compared to preoperative concentrations [44,45], which is associated with worse outcomes and higher mortality [46,47,48]. Glutamine particularly is important in cellular or T cell-induced immune responses, as we found in the first phase of our study.

Basically, T cells are differentiated by the expression of unique cell surface markers, such as CD4+ (helper T cells) and CD8+ (cytotoxic T cells). Various subsets of CD4+ T helper cells are orchestrators of the immune response, since their different subsets may activate a particular immune chain most suitable for fighting a particular threat. One of the most early markers indicating lymphocyte activation is CD69+, which is expressed in almost all subsets of activated T cells. Naive CD4+ T cells differentiate into Th1, Th2, Th17 and Treg cells, with distinct immunological functions [49]. For example, Th1 cells play an important role in directing the cytotoxic CD8+ immune response against intracellular pathogens and tumour cells, whereas Th17 cells are important in fighting bacterial infections. Treg cells play a central role in regulating immune responses and protecting against autoimmunity. 

As mentioned above, our previous study showed that glutamine- and antioxidant-based immunonutrition resulted in a significant increase in the number of CD4+ T cells in low operative risk cardiac surgery patients with intact cellular vitality. To differentiate the cause of an increase in the number of CD4+ T cells, we evaluated additional parameters of the innate immune response, including canonical markers of the systemic inflammatory response, such as the cytokines TNF-α, IL-6 and IL-10, as well as standard serum markers of infection, including PCT and CRP. Several studies [16,17] showed that the nonspecific immune response in patients undergoing cardiac surgery returns to a normal range approximately 5 days after surgery, so we obtained blood samples on the sixth postoperative day in order to exclude this pathway of increased CD4+ T cells in the IN group. Our results showed that there were no significant differences in cytokine concentrations between the patient groups. Furthermore, there were no significant differences in PCT and CRP concentrations. It is noteworthy that the CRP concentration was elevated in both groups similarly considered as a residual expression of the systemic inflammatory response caused by surgery, and CBP when the specific bacterial inflammatory marker PCT was in normal range. Most studies [50,51,52,53] confirmed that PCT is a highly specific marker of bacterial infection, with a sensitivity and specificity of 86.7% and 85.3%, respectively. Therefore, it was chosen to exclude bacterial infection in our study.

Another focus of our study was to investigate whether elevated counts of CD4+ T cells were associated with their activation status. As an activation marker, we chose CD69+ because of its early expression on all subtypes of T cell surface membranes after activation caused by a particular trigger. Furthermore, we decided to determine this marker considering that, in the absence of an increase in CD69+ expression, it can be assumed that no further differentiation of T cells into their subtypes occurred. CD69+ is a membrane receptor with a very low expression in resting T lymphocytes, but it is rapidly upregulated upon T cell activation [19]. CD69+ exerts a complex regulatory effect on the immune system, affecting the differentiation of T cells, synthesis of cytokines, and modulating the inflammatory response. Our results showed that there was no significant difference between the groups after the intervention on the CD69+ survey.

To sum up, we rejected the hypothesis that the increase in CD4+ T cells may have been associated with septic or aseptic systemic inflammatory response syndrome. Hence, our results imply that immunonutrition based on glutamine and antioxidants may influence an increase in the CD4+ T cell count in the body that could probably enhance their migratory potential, thereby increasing their ability to recognize potential threats requiring the involvement of cellular adaptive immune responses. This may significantly impact the outcome of such patients in cases of bacterial or viral infections.

Limitations include a small cohort of patients. Therefore, the immunological data are very scattered. Larger sample surveys are needed. However, it is likely that, with a bigger cohort, the number of excluded patients would increase, mostly due to failure to comply with the dietary rules. We want to emphasize that the results of the study should be regarded as explanatory and exploratory, shedding light on some of the possible immunological mechanisms and ways to promote a proper immune response in cardiac surgery.

## Conclusions

Our findings showed that CD4+ T cell levels were associated with neither the systemic inflammatory response nor bacterial infection. Secondly, increases of T cells are not accompanied by their activation status. These results suggest a hypothesis that a higher postoperative T cell concentration may be associated with postoperative immunonutrition in low-risk cardiac surgery patients. However, immunonutrition alone did not affect T cell activation status.
